# *Saccharomyces cerevisiae* Concentrates Subtoxic Copper onto Cell Wall from Solid Media Containing Reducing Sugars as Carbon Source

**DOI:** 10.3390/bioengineering8030036

**Published:** 2021-03-06

**Authors:** Lavinia L. Ruta, Ileana C. Farcasanu

**Affiliations:** Department of Organic Chemistry, Biochemistry and Catalysis, Faculty of Chemistry, University of Bucharest, Sos. Panduri 90-92, 050663 Bucharest, Romania; lavinia.ruta@chimie.unibuc.ro

**Keywords:** copper, *Saccharomyces cerevisiae*, carbon source, coloration, extracellular deposit

## Abstract

Copper is essential for life, but it can be deleterious in concentrations that surpass the physiological limits. Copper pollution is related to widespread human activities, such as viticulture and wine production. To unravel aspects of how organisms cope with copper insults, we used *Saccharomyces cerevisiae* as a model for adaptation to high but subtoxic concentrations of copper. We found that *S. cerevisiae* cells could tolerate high copper concentration by forming deposits on the cell wall and that the copper-containing deposits accumulated predominantly when cells were grown statically on media prepared with reducing sugars (glucose, galactose) as sole carbon source, but not on media containing nonreducing carbon sources, such as glycerol or lactate. Exposing cells to copper in liquid media under strong agitation prevented the formation of copper-containing deposits at the cell wall. Disruption of low-affinity copper intake through the plasma membrane increased the potential of the cell to form copper deposits on the cell surface. These results imply that biotechnology problems caused by high copper concentration can be tackled by selecting yeast strains and conditions to allow the removal of excess copper from various contaminated sites in the forms of solid deposits which do not penetrate the cell.

## 1. Introduction

Copper is one of the transitional metals that are required in trace amounts for the normal metabolism of living cells and organisms. Copper is required as a cofactor for a variety of enzymes with important roles in cellular processes such as respiration, iron transport, and antioxidant defense, including the universal Cu/Zn superoxide dismutase, and cytochrome c oxidase [1,2,3]. Copper ions shuttle easily between two oxidative states, Cu(I) and Cu(II), a trait exploited by the majority of copper-dependent proteins [3]. Although essential for life, copper can be toxic in concentrations that surpass the physiological thresholds by generating aggressive free radicals via the Fenton reaction or by disturbing protein function through nonspecific binding and structure alterations [4,5]. To elude the deleterious action of surplus copper, organisms have evolved intricate mechanisms to control the transport, homeostasis, and redox buffering [2,6,7]. Defects in copper homeostasis can be associated with a variety of serious human diseases such as Wilson disease, Menkes disease, Parkinson’s, Alzheimer’s, familial amyotrophic lateral sclerosis, copper-dependent anemia, and periodontitis [8,9,10,11,12,13,14,15,16,17,18,19]. Exposure to high levels of copper is another potential copper-related hazard to human health. Copper pollution has various sources, from copper smelting to copper-rich food additives used in animal husbandry and copper-based fungicides used in viticulture [20,21,22]. In fact, copper is unavoidable in winemaking mainly due to the extensive use of cupric pesticides [22,23]. Copper poisoning has many adverse effects such as digestive disorders, neurological disorders, and even atherosclerosis via oxidative stress [24]. There is a rather narrow range between essential and toxic concentrations of copper: it was shown that copper can be deleterious even at low concentrations by altering the immune response, increasing the host susceptibility to pathogens [21,25].

The budding yeast *Saccharomyces cerevisiae* is a eukaryote model often used to investigate various molecular mechanisms that are conserved from yeast to mammals; in this respect, identification of proteins involved in yeast copper metabolism has provided clues for the identification and characterization of mammalian orthologs, many of which, when mutated, lead to human diseases [2]. In this study, we investigated the copper bioaccumulation by yeast cells exposed to sublethal concentrations, noting that copper bioaccumulation grossly depended on the carbon source present in the growth medium.

Copper transport and homeostasis in *S. cerevisiae* have been extensively reviewed [2,26,27]. Under normal growth conditions for yeast, the concentration of copper is variable, from approximately 0.1 µM in synthetic media to a maximum of 2–5 µM in rich media [28]. At such low concentrations, copper is taken up by the high-affinity Cu(I) transporter, Ctr1, following the Cu(II) reduction to Cu(I) by the cell surface metalloreductases Fre1 and Fre2. The expression of *CTR1* and *FRE1* genes is upregulated by copper deficiency via the transcription factor Mac1 [29,30,31]. Ctr1 has a Km for copper of approximately 1–5 µM. Under higher copper concentrations (usually >5 µM), Ctr1 undergoes degradation at the cell surface and its gene expression is suppressed [31], conditions under which copper is taken up by metal transporters with low affinity for copper, namely Fet4, Smf1, or Pho84 [32,33,34,35].

It was shown that under growth conditions that favor respiration (by replacing fermentable glucose with the nonfermentable glycerol), surplus copper increased the life span of yeast cells [36]; in fact, redox-active metals such as copper and manganese were shown to alleviate oxidative stress under nonfermentative conditions [37,38]. Understanding the copper-yeast relation is also relevant from a biotechnology angle, since copper exposure of yeast can be a problem during wine production. Cupric compounds have been extensively used in viticulture to protect grapes against various pests (e.g., Bordeaux mixture), and hence grapes used for wine can have high copper content. In winemaking, *S. cerevisiae* is used to complete the alcoholic fermentation and also to remove the redundant copper ions if the copper concentration is low enough not to interfere with yeast metabolism [22,23]. The presence of copper in the fermentation medium can induce a rust coloration of yeast strains due to the reaction with hydrogen sulfide produced by yeast cells, a significant problem in the wine industry [39,40,41].

To further exploit yeast as a model for copper-related metabolism, we investigated the role of carbon source on the copper accumulation by yeast cells, and we found that reducing sugars such as glucose and galactose favor precipitation of copper at the cell wall level in the form of cuprous compounds.

## 2. Materials and Methods

### 2.1. Growth Media and Yeast Strains

Yeast strains were pre-grown and maintained between experiments in rich medium, YPD (1% w/v yeast extract, 2% w/v peptone, 2% w/v glucose), or synthetic complete SC (0.67 % w/v yeast nitrogen base with (NH_4_)_2_SO_4_, 2% w/v glucose, supplemented with the necessary amino acids) [28]. Experiments involving exposure to copper were done in minimal defined medium (MM) [28] containing 2% w/v glucose (MM/Glc), 2% w/v galactose (MM/Gal), 2 w/v % fructose (MM/Fru), 2% w/v sucrose (MM/Suc), 2% w/v maltose (MM/Mal), 4% v/v glycerol (MM/Gly), or 1% w/v lactate (MM/Lac). MM used had trace metal concentrations of 2 µM MnCl_2_, 2 µM ZnCl_2_, 1 µM FeCl_3_ [42], and 10 μM CuCl_2_ to repress high-affinity copper uptake [31]. For solid media, 2% w/v agar was used. Surplus copper was added to the media from sterile CuCl_2_ stocks after autoclaving and cooling to 60 °C.

For copper-depleted media (MM − Cu), CuCl_2_ was omitted from the recipe, and the medium was supplemented with 5 µM bis(cyclohexanone)oxaldihydrazone (Cuprizone, a Cu(II) chelator) and 5 µM bathocuproine disulfonate (BCS, a Cu(II) chelator) for removal of eventual copper traces.

The *S. cerevisiae* strains used in this study were isogenic to strain BY4741 (*MAT***a**, *his3Δ1*, *leu2Δ0*, *met15Δ0*, *ura3Δ0*). The knockout strains had the genotype BY4741 *orf::kanMX4*, where the gene open reading frame (*ORF*) had been replaced by *kanMX4*. The strains were obtained from EUROSCARF and are designated *orfΔ* throughout the manuscript. These strains were *fet4Δ*, *smf1Δ*, and *pho84Δ*.

Unless otherwise specified, all reagents used were purchased from Merck (Darmstadt, Germany).

### 2.2. Cell Growth on Copper-Supplemented Media

#### 2.2.1. Growth on Solid Media

Overnight pre-cultures were washed and diluted in fresh MM medium at a density of 5 × 10^5^ cells/mL and then grown for 2–4 h at 28 °C with agitation (200 rpm). The exponentially growing cells were 10-fold serially diluted in MM medium and spotted onto agar plates (5 µL/spot). Plates were photographed after 3–9 days of incubation at 28 °C in the dark.

#### 2.2.2. Growth in Liquid Media

Overnight pre-cultures were inoculated in MM at a density of 5 × 10^5^ cells/mL. Cells were incubated for 2–4 h with shaking (200 rpm) at 28 °C on a multiamplitude orbital constant temperature shaking incubator (Thermo Max Q 4000, Thermo Fisher Scientific, Marietta, OH, USA) prior to various tests. The cell density in liquid media was monitored at time intervals by determining the turbidity of the cellular suspension at 660 nm [43] recorded in a plate reader equipped with a thermostat and shaker (Varioskan, Thermo Fisher Scientific, Vantaa, Finland).

### 2.3. Copper Assay

#### 2.3.1. Manipulation of Cells Loaded with Copper on Solid Plates

Patches of cells grown as described in Section 2.2.1 were harvested by means of a Teflon blade (without scratching the agar surface), placed in a centrifuge filter unit (PVDF membrane, 0.45 µm, sample volume up to 0.5 mL, Millipore, Merck, Darmstadt, Germany), and centrifuged (10,000 rpm, 3 min) to remove excess extracellular liquid and loosely cell-bound molecules. Cells were washed with 1 mM 2-(*N*-morpholino)ethanesulfonic acid (MES)–Tris buffer, pH 6.0, containing 5 mM ethylenediaminetetraacetic acid (EDTA, tetrasodium salt, to remove extracellular cations), re-suspended in deionized water, and immediately subjected to total protein and total cell-bound copper assays.

#### 2.3.2. Manipulation of Cells Loaded with Copper in Liquid Media

Yeast pre-cultures were diluted in fresh MM to a density of 5 × 10^5^ cells/mL. The cells were incubated with shaking for 2–4 additional hours at 28 °C before CuCl_2_ was added from sterile stocks. For copper accumulation assay, cells were harvested at various times by centrifugation and washed three times with MES–Tris buffer, pH 6.0, containing 5 mM EDTA. Cells were finally suspended in deionized water and immediately subjected to total protein and total cell-bound copper assays. In a parallel experiment, cells were exposed to copper in liquid media under static conditions (28 °C, no agitation).

#### 2.3.3. Measuring Cell Wall Copper Release

This was done as described by Wofford et al. [44], with slight modifications. Yeast cells loaded with copper were resuspended in 1.2 M sorbitol in 1 mM MES–Tris buffer, pH 6.0, which contained 0 or 10 U/µL lyticase (Zymolyase 100T, USBiological, Salem, MA, USA). Following incubation at 30 °C, cell wall degradation and spheroplast formation were checked by optical microscope (approximately 30 min). Spheroplasts were harvested by centrifugation (800 rpm, 5 min), and the supernatant was collected for the determination of copper released from the cell wall.

#### 2.3.4. Total Cellular Copper Assay

Cells, protoplasts, or supernatants were digested for 24 h with 65% HNO_3_ of ultrapure grade (Merck, Darmstadt, Germany) and stabilized in Tris/HCl buffer (pH 8). Apart from cell digestion and degradation of organic material, HNO_3_ ensures the complete oxidation of Cu(I) to Cu(II). The copper released by cell digestion was assayed colorimetrically with the chromogenic reagent bis(cyclohexanone)oxaldihydrazone (Cuprizone) [45,46] and normalized to cell total protein [47] as nmol copper/mg cell protein or to 10^8^ cells.

### 2.4. Microscopy

To visualize cells grown on agar plates, cells were picked with a sterile loop and gently suspended in 5 μL sterile 10 mM MES–Tris buffer, pH 6.8, directly on the microscope slide. To visualize Cu(I) bound to the cell surface, cells were suspended in 5 μL 10 mM MES–Tris buffer, pH 6.8, containing 1 mM bathocuproine disulfonate (BCS). BCS is a cell-impermeant chelator of Cu(I) with which it forms a blue complex. Bright-field observation of cells was done with an Olympus microscope system (BX53, Olympus, Japan) equipped with an Olympus DP73 camera (Olympus, Tokyo, Japan). Dark-field light scattering by cells or by Cu_2_O particles was monitored using a dark-field condenser and a 100-W halogen lamp. The microscopic photographs were processed by CellSens Dimension V1 imaging software (Olympus, Tokyo, Japan). For each strain, one representative image is shown.

### 2.5. UV–Vis Spectra of Copper-Loaded Cells

Cells grown for 6 days on solid MM containing 0 or 0.1 mM CuCl_2_ were harvested as described in Section 2.3.1, resuspended in deionized water, and frozen at −45 °C to allow cell breakage and release of cellular soluble content. This step was necessary to eliminate the strong noise signal recorded when using intact cells. After defrosting with gentle agitation, cell ghosts were washed with ice-cold deionized water and resuspended to an equivalent of 10^7^ cells/mL. The UV–Vis spectra of yeast cell ghosts were recorded in a quartz cuvette using a UV–Vis spectrophotometer (Jasco V 630, JASCO Corporation, Tokyo, Japan). Cuprous oxide (Cu_2_O) was used as a nonsoluble Cu(I) control: Cu_2_O powder was suspended (1 mg/mL) with strong agitation in deionized water and filtered through a 5 μM membrane to remove the large-size particles. The filtrate was allowed to settle for 2 min and the resulting suspension was used to record the UV–Vis spectrum.

### 2.6. Reproducibility of the Results and Statistics

All experiments were repeated at least three times. For visual data, one representative example is shown. For each individual measurement, values are expressed as the mean ± standard error of the mean (SEM). The data were examined by analysis of variance with multiple comparisons (ANOVA) using the statistical software Prism version 6.05 for Windows (Version 5.03, GraphPad Software, La Jolla, CA, USA). One sample *t*-test was used for the statistical analysis of each strain/condition compared with a strain/condition considered as reference. The differences were considered to be significant when *p* < 0.05.

## 3. Results

### 3.1. Yeast Cells Grown on Media Containing Reducing Sugars Accumulate Copper at the Cell Wall

**Figure 1:**
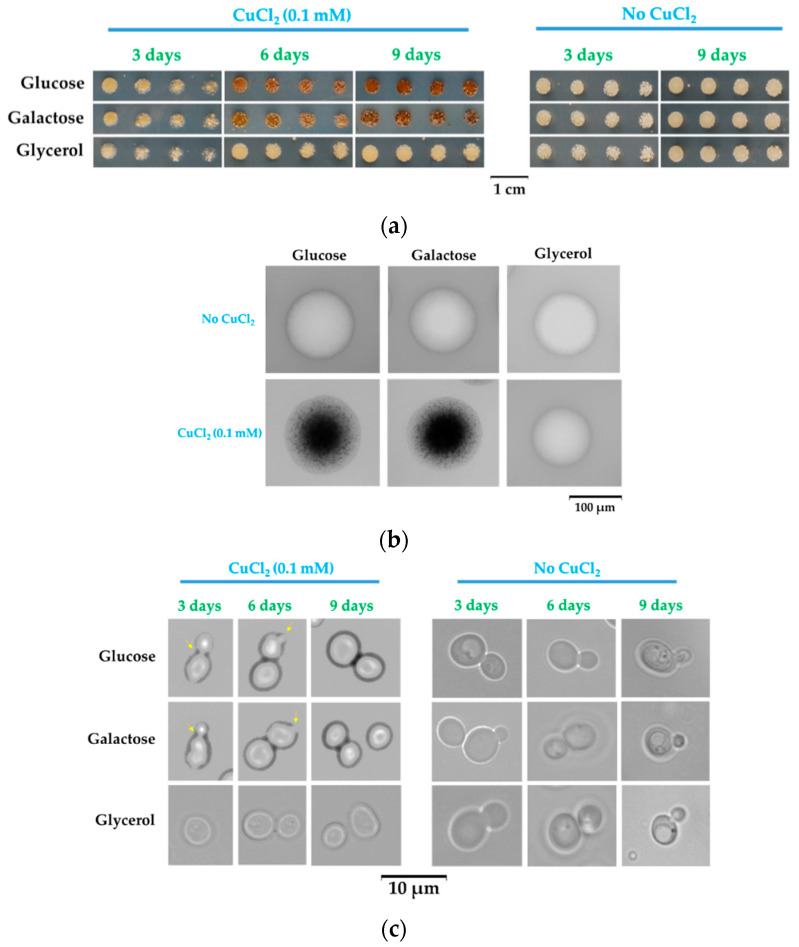
Coloration of *Saccharomyces cerevisiae* grown on copper-containing medium. (**a**) Cell suspensions of strain BY4741 were spotted in serial dilution on minimal defined media (MM)/Glc (Glucose), MM/Gal (Galactose), or MM/Gly (Glycerol) media with or without additional CuCl_2_. Plates were observed daily and photographed at various times. (**b**) Microscopic images of yeast colonies grown from one single cell for 9 days on MM with or without 0.1 mM CuCl_2_. (**c**) Microscopic images of yeast cells grown on media enriched with 0.1 mM CuCl_2_. Cells were picked from duplicate plates shown in (**a**). Grey images are shown, for clarity, in (**b**,**c**).

Under standard growth conditions, copper concentration in yeast media is 0.1–5 μM [28], but when cultivated on agar plates, yeast cells can easily grow on media that contain up to 1 mM, which is far above the normal threshold. This apparent lack of toxicity is the result of copper being buffered by the multitude of ligands that coexist in the yeast growing medium (e.g., amino acids, phosphate), keeping the concentration of free ions rather low. Nevertheless, it is expected that cells grown on copper-enriched media would have a dynamic and continuous exchange of ions with the medium. When testing various conditions in which yeast cells tolerate increased copper concentrations, we noticed that growing yeast cells on solid media containing subtoxic concentrations of copper resulted in cell coloration, usually from creamy white to brown, and this was noticed on both rich medium (YPD) and synthetic media (SC or MM). In all our subsequent experiments, we used the minimal defined media (MM) as they have controlled and reproducible chemical composition [28]. On MM/agar media, wild-type yeast cells could grow in the presence of surplus copper of up to 1.5–2 mM without signs of severe toxicity. However, we noticed that under nontoxic copper surplus the color developed by cell populations depended on the carbon source used in the medium recipe. That is, cells grown on media containing glucose turned rusty after 2–3 days of copper exposure; the color intensity increased with exposure time (Figure 1a). The lowest CuCl_2_ concentration for which the change of color was detectable by naked eye was 0.05–0.1 mM for MM.

When changing glucose with galactose—another reducing sugar often used in experimental yeast media—the cells also changed color in time (Figure 1a, middle row). In contrast, when the carbon source was glycerol, the cells no longer changed color during copper exposure (Figure 1a, bottom row), suggesting that reducing sugars may play a role in cell coloration. Microscopically, the yeast cell populations grown on agar plates containing copper appeared with dark spots on their surface when grown on reducing sugars glucose and galactose; such spots were absent on cell populations grown on glycerol media (Figure 1b). Overall, the colonies obtained from single cells plated on MM/agar media appeared dark brown when copper exposure was done in the presence of glucose or galactose, being darker in the center, where the majority of cells accumulated (Figure 1b); in contrast, when copper exposure was done in the presence of glycerol, the colonies remained uncolored, as in the case of cells not exposed to copper (Figure 1b). Visually, single colonies increased in size up to day 3–4 from plating; afterward, the size of the colonies did not significantly change, but the color of colonies grown on glucose or galactose continued to amplify, becoming dark after 8–9 days of incubation (Figure 1b). When analyzing individual cells, it was noted that cells exposed to copper showed a dark halo when cells were grown on glucose or galactose, and this halo was absent (or very thin) on cells grown on glycerol (Figure 1c, left panel). After approximately 3 days, cells enter the stationary phase. Nevertheless, it was noticed that some cells exposed to copper still proliferated after 3–6 days of low copper exposure in the presence of glucose or galactose (Figure 1c, yellow arrow); this proliferation could no longer be seen after 9 days of exposure, indicating that the cells may have entered a dormant state [44].

### 3.2. Yeast Cells Grown on Solid Media Containing Reducing Sugars as Carbon Source Accumulate Cu(I) at the Cell Wall

**Figure 2:**
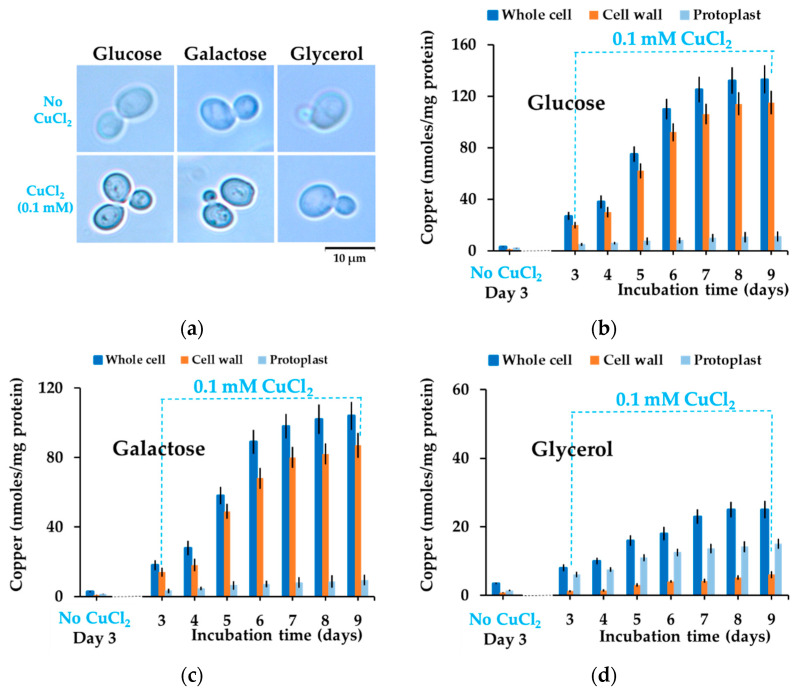
Accumulation of copper by *Saccharomyces cerevisiae* cells exposed to CuCl_2_ on solid media. Exponentially growing cells of wild-type strain BY4741 were spotted (5 μL of 1 × 10^6^ cells/mL suspensions) on agar plates containing MM/Glc (Glucose), MM/Gal (Galactose), or MM/Gly (Glycerol) with or without 0.1 mM CuCl_2_. (**a**) Visualization of copper bound to yeast cell surface. Cells grown on agar plates for 6 days were suspended in 5 µL of buffer containing 0.1 mM bathocuproine disulfonate (BCS) directly on microscope slide. Cells were visualized in bright field 10 minutes after BCS staining. (**b**–**d**) Copper accumulation by cells grown statically on solid agar media and its distribution between cell wall and protoplasts. Cell suspensions were spotted on agar plates as in (**a**) and incubated in a dark incubator. Starting from day 3, cell patches were harvested and processed for copper assay, as described in Section 2. Cells from the same batch in MM/Glc (**b**), MM/Gal (**c**), or MM/Gly (**d**), were used to determine whole-cell copper, cell wall copper, or copper accumulated in protoplasts. Values were normalized to cell total proteins in the corresponding intact cells. Values are mean ± SEM of triplicate determinations done on three biological repeats.

It was shown that yeast cells change color and turn brown in the presence of copper due to the mineralization of copper sulfide at the cell surface [39,40,41]. The main gene responsible for this phenotype is thought to be *SLF1*, as *slf1Δ* knockout cells were reported to remain white in the presence of copper [39]. We found that under nontoxic copper exposure, *slf1Δ* cells actually turned brown similarly to wild-type cells. This phenotype occurred only in the presence of the reducing sugars galactose or glucose but not in the presence of glycerol, suggesting a role of reducing sugars in cell coloration, apart from previously reported copper sulfide deposition [39,40,41]. Aldoses are known to reduce Cu(II) to Cu(I), yielding insoluble cuprous compounds (predominantly cuprous oxide); therefore, we speculated that the dark spots on yeast colonies (Figure 1b) or the dark halo covering yeast cells grown on glucose or galactose (Figure 2c) would actually be cell wall deposits containing Cu(I). To test this possibility, we stained the copper-exposed cells with bathocuproine disulfonate (BCS), a cell-impermeant chelator that forms a blue complex with Cu(I). For this purpose, we suspended cells that developed a brown color when grown on copper-containing media (from plates similar to those shown in Figure 1a) in a buffer that contained BCS and observed the cells microscopically. It was noted that cells grown on agar MM/Glc or MM/Gal containing subtoxic concentrations of CuCl_2_ were stained dark blue at the cell surface, indicating that the halo surrounding the copper-exposed cells may indeed contain Cu(I). In contrast, cells exposed to copper in MM/Gly were not stained by BCS at the cell wall, similarly to cells grown in the absence of supplementary copper (Figure 2a).

We next measured the copper bound by cells grown on copper agar plates and found that cells grown on glucose or galactose accumulated significantly more copper than the cells grown on glycerol (Figure 2b–d, blue bars). As copper seems to accumulate at the cell wall level (Figure 1c and Figure 2a), we determined the amount of copper released from cell walls as a result of whole-cell treatment with lyticase, which degrades the polysaccharides that form the cell wall in yeast [44]. We found indeed that most of the copper accumulated by intact cells grown on glucose or galactose came from cell wall deposits (Figure 2b,c, red bars), accounting for the majority of copper accumulated by the cells, in contrast to cells grown on MM/Gly plates, which retained little copper at the cell wall (Figure 2d, red bars). Internalized copper—as determined by measuring total copper associated with protoplasts released after cell wall degradation—was not significantly different for cells grown on either carbon source (Figure 2b–d, light blue bars).

### 3.3. Copper Accumulation by Yeast Cells Grown Statically in Liquid Media

**Figure 3:**
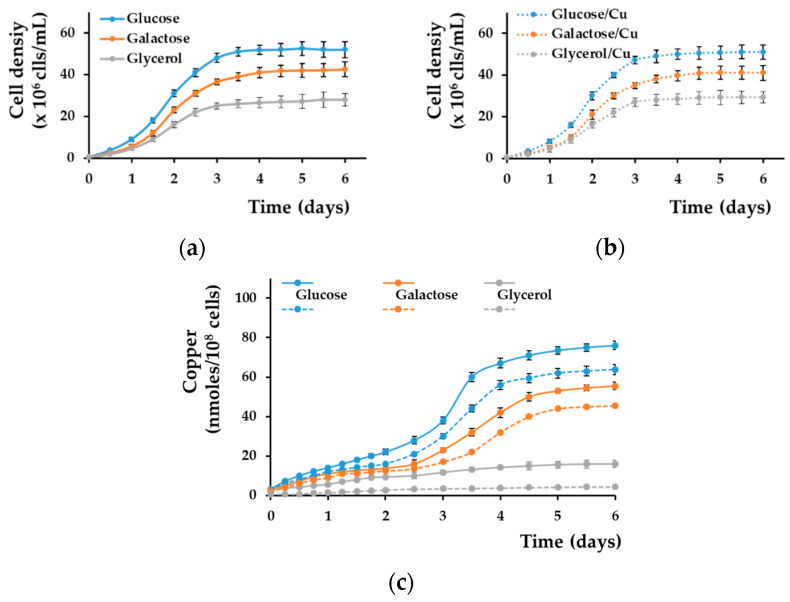
Copper accumulation by yeast cell grown statically in MM liquid media. Cell suspensions of wild-type strain BY4741 were inoculated (5 × 10^5^ cells/mL) in MM/Glc (Glucose), MM/Gal (Galactose), or MM/Gly (Glycerol), and cell proliferation was determined spectrophotometrically (OD_660_) in the absence (**a**) or presence (**b**) of 0.1 mM CuCl_2_. (**c**) Copper associated with whole cells (full lines) or with cell walls (dotted lines) was determined as described in Section 2. Values are mean ± SEM of triplicate determinations done on three biological repeats.

As the high accumulation of copper by yeast cells grown on glucose or galactose compared with glycerol was the result of copper deposition at the cell wall when cells were cultivated statically on the surface of solid media containing copper (Figure 2), we wondered if the same situation occurred when cells were grown submerged in liquid media. Cells grown statically in liquid MM reached the stationary phase after approximately 3 days of incubation, both in the absence and in the presence of nontoxic copper (Figure 3a,b). Copper accumulation could be observed in both exponential and stationary phases, significantly increasing when shifting from exponential to stationary growth (days 2–4) for cells grown in MM/Glc or MM/Gal (Figure 3c, blue and red full lines, respectively). Overall, copper accumulation reached a plateau after 6 days of incubation. In contrast, copper accumulation from MM/Gly was not significant in the stationary phase (Figure 3, grey full line).

The majority of copper accumulated by cells from MM/Glc or MM/Gal media prevailed at the cell wall level (Figure 3c, blue and red dotted lines, respectively). In contrast, copper bound to the wall of cells grown in MM/Gly was much lower (Figure 3c, dotted grey line). Apparently, static conditions are needed for copper deposition at the cell wall, as incubating cells with agitation (200 rpm) resulted in a lower occurrence of copper at the cell wall level. Under strong agitation, copper deposition at the cell wall was hampered, and the copper accumulation was not significantly different for cells grown in MM/Glc, MM/Gal, or MM/Gly (Figure S1).

**Figure 4:**
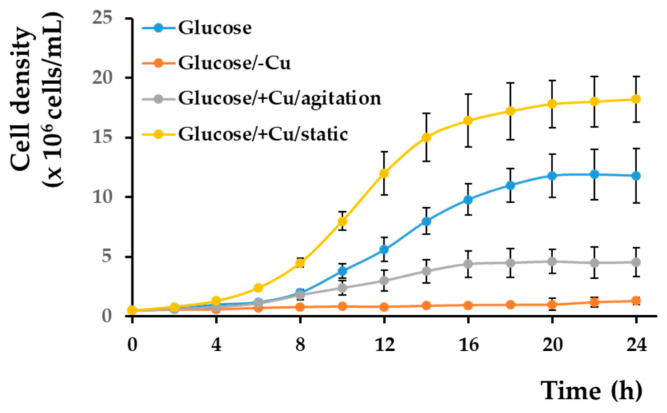
Growth of copper-loaded and copper-starved cells in copper-deficient medium. Wild-type strain BY4741 was pre-grown for 16 h in liquid MM/Glc (Glucose), MM/Glc without Cu (glucose/–Cu), or MM/Glc with 0.1 mM CuCl_2_ under agitation (Glucose/+Cu/agitation) or for 3 days statically in MM/Glc with 0.1 mM CuCl_2_ (Glucose/+Cu/static) before being washed and shifted to MM/Glc/–Cu at density 5 × 10^5^ cells/mL. Cell proliferation was determined spectrophotometrically (OD_660_) as described in Section 2. Values are mean ± SEM of triplicate determinations done on three biological repeats.

It was reported that iron is deposited in the cell wall when the cells reach the stationary phase, but it is mobilized when the cells start growing again [44]; therefore, we wondered if a similar situation would occur in the case of cells with copper deposits. To check this possibility, we shifted cells pre-grown in various copper conditions to copper-depleted MM/Glc and checked the cell proliferation. We noticed that cells pre-grown statically in a copper-containing medium could grow very well when shifted to copper-depleted medium compared to cells pre-grown in standard MM, and their growth was significantly better than that of the copper pre-starved cells (Figure 4). This observation suggests that cells pre-incubated with copper can utilize the copper accumulated at the cell wall to compensate for the absence of this metal in copper-deficient environments. In this line of evidence, it was noted that cells pre-incubated with copper under strong agitation (when copper deposition at the cell wall is prevented, Figure S1) did not proliferate to the same extent when copper deposition at the cell wall level was prevalent as the cells pre-grown statically in the presence of copper (Figure 4).

### 3.4. The Coloration of Yeast Populations Fades when Copper Concentration Increases

**Figure 5:**
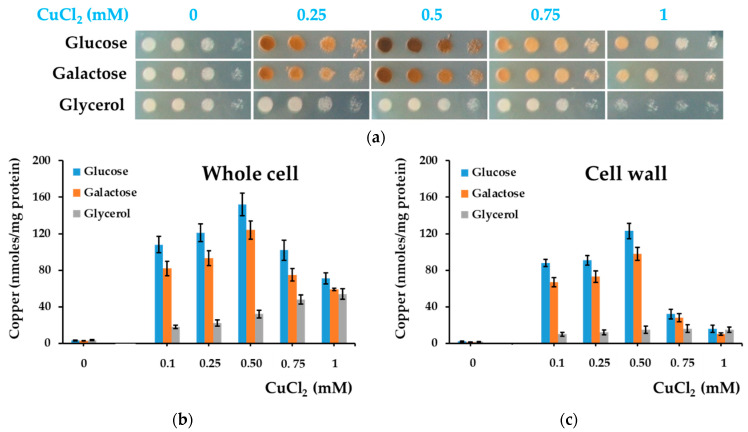
Coloration of *Saccharomyces cerevisiae* and accumulation of copper at higher copper concentrations. (**a**) Cell suspensions of strain BY4741 were spotted in serial dilution onto MM/Glc (Glucose), MM/Gal (Galactose), or MM/Gly (Glycerol) media with various concentrations of CuCl_2_. Plates shown were photographed after 6 days of incubation at 28 °C. (**b**) Copper accumulation by cells grown statically on MM agar media. Cell suspensions were spotted on agar plates (approximately 10^6^ cells/5 µL spot) and incubated at 28 °C. (**c**) Copper associated with cell walls. Cell patches were harvested after 6 days of incubation and processed for copper assay, as described in Section 2. The cells used to determine whole-cell copper, cell wall copper, and total cell protein used for normalization belonged to the same batch. Values are mean ± SEM of triplicate determinations done on three biological repeats.

It was surprising to notice that the copper-related coloration of yeast populations started to fade when increasing the copper concentration in the medium (Figure 5a), probably due to inhibition of copper deposition on the cell surface. To test this possibility, we measured copper bound to yeast cells exposed to increasing copper concentrations (Figure 5b).

It was noted that the amount of total copper accumulated by yeast cells from solid media was high when cells were grown on MM/Glc or MM/Gal agar media in comparison to copper accumulation by cells grown on MM/Gly agar media, but only for concentrations in the range 0.1–0.5 mM. At copper concentrations higher than 0.5 mM, the total copper bound by yeast cells grown on agar MM/Glc or MM/Gal gradually decreased to finally reach a value that was not significantly different from the case when cells were grown on MM/Gly (Figure 5b). It was also noted that copper associated with the cell wall decreased with increasing copper concentration in the MM/Glc or MM/Gal media, accounting for the fading of the rust color when copper concentration increased. This observation suggests that higher concentrations of copper in the growth media inhibit the copper deposition at the cell wall, and this can be visualized directly by the loss of cell color. It is possible that exposure to high copper concentration induces glucose/galactose mobilization for various cell defense mechanisms, leaving lower levels of reducing sugars available for copper deposition. Alternatively, it is possible that high copper concentrations induced modification in the processes of cell wall synthesis which do not favor copper deposition in the cell wall.

### 3.5. Cells Defective in Fet4—A Low-Affinity Copper Transporter—Accumulate More Copper on the Cell Surface

**Figure 6:**
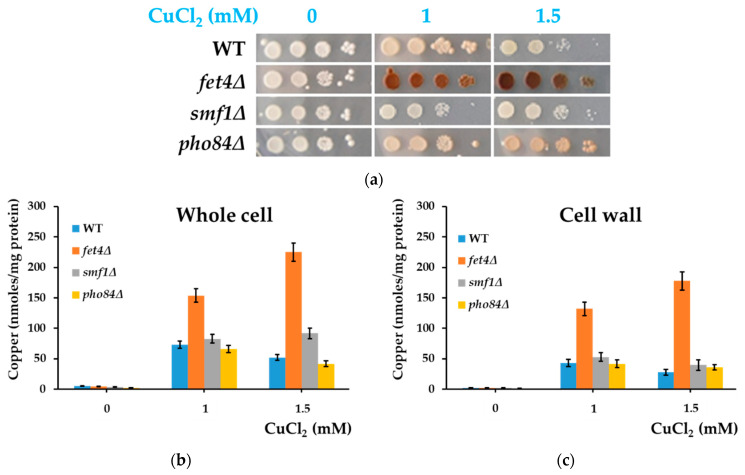
Coloration and accumulation of copper by *Saccharomyces cerevisiae* strains with defects in low-affinity transport of copper across the plasma membrane. (**a**) Cell suspensions of strain BY4741 (WT) and of isogenic strains *fet4Δ*, *smf1Δ*, and *pho84Δ* were spotted in serial dilution onto MM/Glc (Glucose) media with various concentrations of CuCl_2_. Plates shown were photographed after 6 days of incubation at 28 °C. (**b**) Copper accumulation by cells grown statically on MM/Glc agar media. Cell suspensions were spotted on agar plates (approximately 10^6^ cells/5 µL spot) and incubated at 28 °C. Cell patches were harvested after 6 days of incubation and processed for copper assay, as described in Section 2. (**c**) Copper associated with cell walls. The cells used to determine whole-cell copper, cell wall copper, and total cell protein used for normalization belong to the same batch. Values are mean ± SEM of triplicate determination done on three biological repeats.

As increased copper concentrations decreased the overall copper accumulation by yeast cells, probably by inhibiting the deposition of copper compounds onto the cell surface, we wondered what would happen when the low-affinity copper transport into the cell was perturbed. All our experiments done in this study employed media that had subtoxic but higher-than-usual concentrations of copper in the culture media. Under such conditions, the high-affinity Ctr1 copper transporter is repressed [31] and copper is taken up by cells via low-affinity transporters. We, therefore, tested if yeast cells with defects in the low-affinity transport of copper across the plasma membrane would exhibit changes in copper-related coloration or accumulation. There are three transporters known to have a minor role in the low-affinity transport of copper: Fet4, Smf1, and Pho84 [30,31,32,33]. We noted that the knockout *fet4Δ* formed cell populations that turned dark brown even at high copper concentrations (1.5 mM), conditions under which WT and *smf1Δ* remained white and *pho84Δ* was slightly colored (Figure 6a).

We measured the cell-bound copper in the cell populations harvested from the agar plates and found that *fet4Δ* cells accumulated the highest amount of copper as related to whole cells (Figure 6b). This apparent paradox suggested that the high amount of copper recorded for *fet4Δ* cells grown on agar plates was the result of copper precipitation onto the cell surface. Indeed, *fet4Δ* cells exhibited the highest distribution of copper at the cell wall level (Figure 6c). Interestingly, when cells were exposed to copper in liquid medium under strong agitation (when copper deposition at the cell surface is not favored) the total copper associated with whole cells was lowest in the case of *fet4Δ* cells (Figure S2), indicating that the high level of copper bound to *fet4Δ* under static incubation is indeed the result of copper deposition at the cell wall.

It was intriguing to note the difference between the three knockout mutants in terms of copper-induced coloration. Fet4 is a low-affinity ferrous ion transporter also involved in copper transport, preferring cations in lower oxidation state [32]; under such conditions, it is possible that *fet4Δ* would keep out an excess of Cu(I) compared to *smf1Δ* and *pho84Δ*, hence the stronger coloration of *fet4Δ* in the presence of high copper content. It was suggested that the difference in cell coloration was the result of growth defects, but no significant difference between the growth of *fet4Δ*, *smf1Δ*, and *pho84Δ* could be noted (Figure S3).

### 3.6. Copper Deposits at the Cell Wall Contain Cuprous Oxide

**Figure 7:**
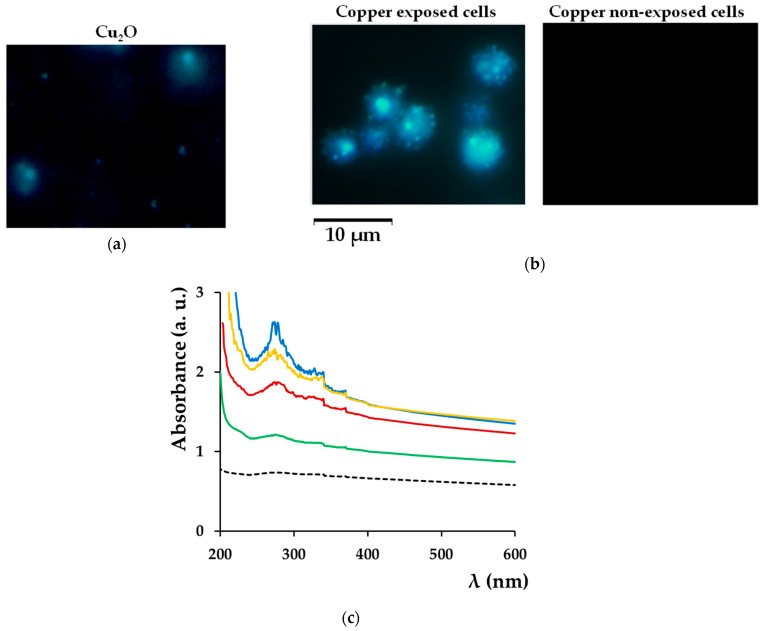
Optical properties of copper-exposed cells. Light scattering by Cu_2_O particles (**a**) or by cells exposed to copper (**b**). BY4741 cells were grown for 6 days on MM/Glc with or without 0.1 mM CuCl_2_ before being observed microscopically in dark field. (**c**) UV–Vis spectra. Cells were processed as described in Section 2. Spectra correspond to cells grown for 6 days on solid MM/Glc (blue line), MM/Gal (Yellow), or MM/Gly (green) containing 0.1 mM CuCl_2_ or in MM/Glc without CuCl_2_ (black dotted line). Spectrum of Cu_2_O particles (red line) was included as control.

Reducing sugars, such as glucose, are known to reduce Cu(II) to Cu(I) yielding cuprous oxide (Cu_2_O), a rusty-brown precipitate; therefore, we wondered if the copper deposits formed at the yeast cell wall in media prepared with reducing sugars contained Cu_2_O. The Cu_2_O particles can be visualized microscopically in dark field, as they have light-scattering properties [48]. We noticed that—similarly to Cu_2_O particles (Figure 7a)—cells exposed to copper in MM/Glc also scattered light (Figure 7b), indicating that Cu_2_O may form at the cell wall level. To test this possibility we compared the UV–Vis spectra recorded for yeast cells exposed to copper with the UV–Vis spectrum of a suspension containing Cu_2_O particles. We found that spectra corresponding to cells exposed to copper in MM/Glc or MM/Gal media showed similar traces when compared with the Cu_2_O spectrum (Figure 7a,b), supporting the idea that the presence of reducing sugars in the incubation media facilitated copper deposition in the form of Cu(I) compounds, including Cu_2_O.

### 3.7. The Influence of Other Carbon Sources on Formation of Copper Deposits

**Figure 8:**
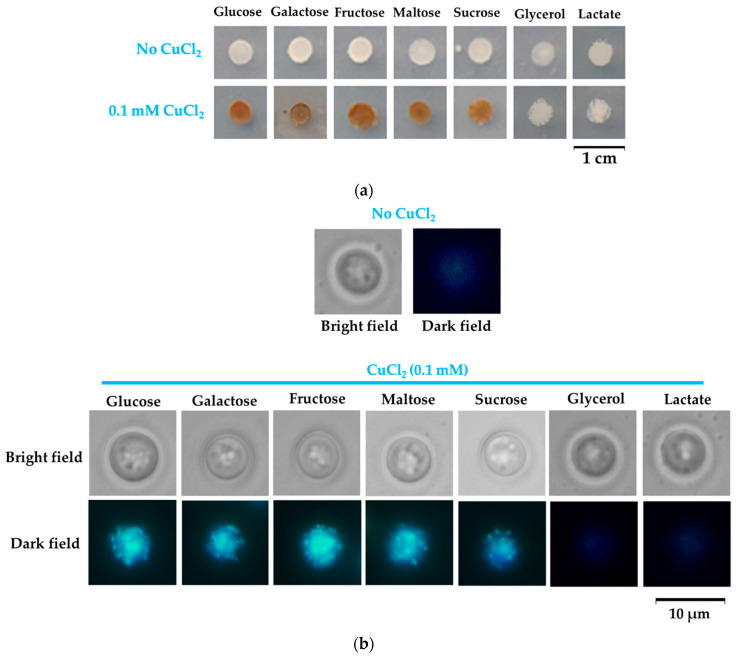
Influence of carbon source on copper deposition on the *Saccharomyces cerevisiae* cells. (**a**) Cell suspensions of strain BY4741 were spotted (5 μL containing approximately 10^4^ cells initially) onto MM/Glc (Glucose), MM/Gal (Galactose), MM/Fru (Fructose), MM/Mal (Maltose), MM/Suc (Sucrose), MM/Gly (Glycerol), or MM/Lac (Lactate) media with or without 0.1 mM CuCl_2_. Plates were photographed after 6 days of incubation at 28 °C. (**b**) Microscopic images of yeast cells isolated from cell population shown in (**a**). Cells were picked by a sterile loop, washed gently, and suspended in 5 μL water directly in the microscope slide. For bright-field images, automatic time exposure was used (an average of 1 ms). For dark-field images, exposure time was 1 s.

The observation that cells turn rusty-brown in the presence of the reducing sugars glucose and galactose, but not in the presence of the nonreducing glycerol, prompted the necessity to test other compounds to see if the coloration can be indeed correlated to the reducing traits of the carbon source. We, therefore, exposed yeast cells to copper added to MM which contained fructose, sucrose, maltose, or lactate as the sole carbon source. We noticed that copper exposure resulted in cell coloration in the case of all carbon sources, except glycerol and lactate (Figure 8a).

The color of the cell populations could be easily explained for maltose, as it is a reducing disaccharide, while the noncoloration of cells grown on lactate (a nonreducing substrate) was also as expected. Fructose is not a reducing sugar, but it can easily epimerize to glucose, inducing cell coloration. Sucrose is a nonreducing disaccharide, and therefore we would have expected noncolored cells; the presence of the rusty color can be explained by the fact that sucrose is hydrolyzed to glucose and fructose by the invertase secreted by yeast cells when sucrose is the only carbon source [49]. Since reducing sugars turn Cu(II) ions to Cu(I), often with the formation of cuprous oxide Cu_2_O, we wondered if the copper deposits at the cell wall level may contain traces of Cu_2_O. To check this possibility, we monitored the light-scattering traits of yeast cells exposed to copper in the presence of the carbon sources tested in this study. Metal-containing nanoparticles express unique light-scattering characteristics, and it was shown that Cu_2_O nanoparticles express blue light patterns on bacteria cells [48]. We noticed that yeast cells pre-exposed to copper in media containing reducing sugars as the sole carbon source (or sugars that can generate reducing compounds) expressed blue light in the form of spots; this trait could not be observed in the case of cells grown on nonreducing carbon sources such as glycerol and lactate (Figure 8b).

## 4. Discussion

Although essential for life, copper can be extremely toxic when surpassing the physiological limits, which vary considerably in regard to species and environmental conditions. The toxicity of copper is often alleviated by the co-presence in the environment of various ligands, which buffer its eventual toxicity. Copper shuttles between two oxidation states, Cu(II) and Cu(I), and it is Cu(I) which is considered the much more toxic ion [1,2,3,4]. In fact, Cu(I) is so toxic that some known antioxidants can shift from good to deleterious simply by locally increasing the concentration of Cu(I) [50]. For this reason, a very low concentration of free Cu(I) is maintained within the living cells through a plethora of chemical and physiological mechanisms [51], and the stratagems include oxidation, complexation, chaperoning, and induction of metal buffering proteins such as metallothioneins [1,2,3,4,26,27].

In this study, we showed that the carbon source had a substantial influence on how cells deal with high copper situations. We found that under high but subtoxic copper concentrations, yeast cells still can grow, but they turn brown if the carbon source is a reducing sugar (i.e., glucose or galactose). Apparently, this change in color was the result of copper deposition on the surface of yeast in the form of Cu(I) compounds, as shown by BCS staining (Figure 2a). The copper-containing deposits seemed harmless though, as cells underwent division even under the burden of a thick copper-containing layer (Figure 1c). It had been previously reported that the brown coloration of yeast cells in the presence of copper was the result of copper mineralization in the form of CuS mediated by *SLF1* gene or by genes involved in hydrogen sulfide production [39,40,41], but while the role of sulfide in the brown colorization of yeast cells cannot be ruled out, it became clear that copper depositions at the cell surface also depend on the reducing power of sugars present in the incubation medium. In this line of evidence, we noticed that knockout *slf1Δ* cells still turned brown on MM/Glc or MM/Gal but not on MM/Gly plates, similarly to wild-type cells. Moreover, cells exposed to copper in media containing reducing sugars formed deposits that had optical characteristics similar to Cu_2_O particles (Figure 7 and Figure 8b).

It was surprising to notice that under nontoxic excess, copper accumulated in both exponential and stationary growth phases: this can be explained by the buffering mechanisms induced by copper exposure, such as metallothionein induction (which was shown to require approximately 24 h for full copper saturation [52]) or cell wall modification induced to accommodate solid deposits [44]. That the accumulation rate was higher at the border between exponential and stationary growth could be regarded as the optimal stage when cells become suitably armed—both internally and at the cell wall level—to fight against copper excess. What was intriguing was the cell behavior under high copper content, when not only cell wall deposits were diminished, but even internal copper decreased. It is possible that under high concentrations, the copper ions would dribble by the buffering systems (e.g., metallothionein, cell wall) and cells would turn to emergency defense mechanisms, such as copper extrusion from the cell through excretion or other export mechanisms.

It is not unusual to use media supplements to manipulate cell tolerance to heavy metals, and such supplements can be sugars, amino acids, glutathione, and other antioxidants [53,54,55,56,57,58,59]. As copper can be cumbersome in wine production, choosing the right yeast strain in combination with an optimal concentration of reducing sugars could be beneficial for copper removal from fermentation media. In this regard, it would be interesting to investigate whether the copper deposition at the cell wall reported in this study can be extrapolated to industrial strains. It is tempting to speculate that copper is deposited at the cell wall in the form of Cu_2_O nanoparticles, as suggested by light-scattering traits of cells exposed to nontoxic copper concentrations. Nevertheless, in-depth characterization of these microscopic bodies is needed to establish their chemical composition and the metabolic status of cell wall dynamics necessary for optimal copper deposition at the cell wall.

## 5. Conclusions

Copper can be tolerated by *S. cerevisiae* cells in concentrations that are 2–3 orders of magnitude higher than the physiological levels. Copper binding to the cell surface can be increased considerably by growing the cells statically on media containing reducing sugars as carbon sources. This could represent a valuable tool for the removal of excess copper from different contaminated solids, such as grapes grown in vineyards extensively treated with copper-based pesticides. As yeast cells exposed to copper turn brown in the presence of reducing sugars, they could also find applications as biosensors for the detection of high copper levels in various biological probes.

## Data Availability

Data is contained within the article or supplementary material.

## Supplementary Materials

The following are available online at https://www.mdpi.com/2306-5354/8/3/36/s1, Figure S1: Copper accumulation by yeast cell grown with agitation in MM liquid media. Cells of wild type strain BY4741 were inoculated (5 × 105 cells/mL) in MM/Glc (Glucose), MM/Gal (Galactose) or MM/Gly (Glycerol) and grown with agitation (28 °C, 200 rpm). (a) Cell proliferation was determined spectrophotometrically (OD_660_) in the absence (left panel) or presence (right panel) of 0.1 mM CuCl_2_. (b,c) Copper associated with whole cells (full lines) or with cell walls (dotted lines) was determined as described in Section 2 and normalized to total cell protein (b) or to 10^8^ cells (c). Values are mean ±SEM of triplicate determinations done on three biological repeats; Figure S2: Copper accumulation by yeast mutants grown with agitation (28 °C, 200 rpm) in MM/Glc liquid media. Cell suspensions of strain BY4741 (WT) and of isogenic strains *fet4Δ*, *smf1Δ*, and *pho84Δ* were incubated with 0.5 mM CuCl_2_. Cells were harvested by centrifugation at various times and processed for copper assay, as described in *Materials and methods*. Values are mean ±SEM of triplicate determination done on three biological repeats; Figure S3: Effect of copper on the growth of yeast mutants with defects in metal transport across plasma membrane. Cell suspensions of strain BY4741 (WT) and of isogenic strains *fet4Δ*, *smf1Δ*, and *pho84Δ* were inoculated (5 × 10^5^ cells/mL) in MM/Glc and incubated for 2–4 h (28 °C, 200 rpm) before CuCl_2_ was added to various concentrations. Cell proliferation was determined spectrophotometrically (OD_660_) 24 h following copper addition. Values are mean ± SEM of triplicate determination done on three biological repeats.

## References

[1] Culotta V. (2010). Cell biology of copper. J. Biol. Inorg. Chem..

[2] Nevitt T., Ohrvik H., Thiele D.J. (2012). Charting the travels of copper in eukaryotes from yeast to mammals. Biochim. Biophys. Acta.

[3] Guengerich F.P. (2018). Introduction to metals in biology 2018: Copper homeostasis and utilization in redox enzymes. J. Biol. Chem..

[4] Valko M., Morris H., Cronin M.T. (2005). Metals, toxicity and oxidative stress. Curr. Med. Chem..

[5] Uriu-Adams J.Y., Keen C.L. (2005). Copper, oxidative stress, and human health. Mol. Aspects Med..

[6] Balamurugan K., Schaffner W. (2006). Copper homeostasis in eukaryotes: Teetering on a tightrope. Biochim. Biophys. Acta.

[7] Bleackley M.R., Macgillivray R.T. (2011). Transition metal homeostasis: From yeast to human disease. Biometals.

[8] Bulcke F., Dringen R., Scheiber I.F. (2017). Neurotoxicity of copper. Adv. Neurobiol..

[9] Kelly C., Pericleous M. (2018). Wilson disease: More than meets the eye. Postgrad. Med. J..

[10] Lorincz M.T. (2018). Wilson disease and related copper disorders. Handb. Clin. Neurol..

[11] Ahuja A., Dev K., Tanwar R.S., Selwal K.K., Tyagi P.K. (2015). Copper mediated neurological disorder: Visions into amyotrophic lateral sclerosis, Alzheimer and Menkes disease. J. Trace Elem. Med. Biol..

[12] Bjorklund G., Stejskal V., Urbina M.A., Dadar M., Chirumbolo S., Mutter J. (2018). Metals and Parkinson’s disease: Mechanisms and biochemical processes. Curr. Med. Chem..

[13] Adlard P.A., Bush A.I. (2018). Metals and Alzheimer’s disease: How far have we come in the clinic?. J. Alzheimers Dis..

[14] Hsu H.W., Bondy S.C., Kitazawa M. (2018). Environmental and dietary exposure to copper and its cellular mechanisms linking to Alzheimer’s disease. Toxicol. Sci..

[15] Gil-Bea F.J., Aldanondo G., Lasa-Fernández H., López de Munain A., Vallejo-Illarramendi A. (2017). Insights into the mechanisms of copper dyshomeostasis in amyotrophic lateral sclerosis. Expert Rev. Mol. Med..

[16] Tokuda E., Furukawa Y. (2016). Copper homeostasis as a therapeutic target in amyotrophic lateral sclerosis with *SOD1* mutations. Int. J. Mol. Sci..

[17] Myint Z.W., Oo T.H., Thein K.Z., Tun A.M., Saeed H. (2018). Copper deficiency anemia: Review article. Ann. Hematol..

[18] Dommisch H., Kuzmanova D., Jönsson D., Grant M., Chapple I. (2018). Effect of micronutrient malnutrition on periodontal disease and periodontal therapy. Periodontol. 2000.

[19] Besold A.N., Culbertson E.M., Culotta V.C. (2016). The Yin and Yang of copper during infection. J. Biol. Inorg. Chem..

[20] Wu X., Cobbina S.J., Mao G., Xu H., Zhang Z., Yang L. (2016). A review of toxicity and mechanisms of individual and mixtures of heavy metals in the environment. Environ. Sci. Pollut. Res. Int..

[21] Zhao H., Wang Y., Shao Y., Liu J., Liu Y., Xing M. (2018). Deciphering the ionic homeostasis, oxidative stress, apoptosis, and autophagy in chicken intestine under copper(II) stress. Environ. Sci. Pollut. Res. Int..

[22] Sun X.Y., Zhao Y., Liu L.L., Jia B., Zhao F., Huang W.D., Zhan J.C. (2015). Copper tolerance and biosorption of *Saccharomyces cerevisiae* during alcoholic fermentation. PLoS ONE.

[23] Sun X., Liu L., Zhao Y., Ma T., Zhao F., Huang W., Zhan J. (2016). Effect of copper stress on growth characteristics and fermentation properties of *Saccharomyces cerevisiae* and the pathway of copper adsorption during wine fermentation. Food Chem..

[24] Bui T.K., Do-Hong L.C., Dao T.S., Hoang T.C. (2016). Copper toxicity and the influence of water quality of Dongnai river and Mekong river waters on copper bioavailability and toxicity to three tropical species. Chemosphere.

[25] Soedarini B., Klaver L., Roessink I., Widianarko B., Van Straalen N.M., Van Gestel C.A. (2012). Copper kinetics and internal distribution in the marbled crayfish (*Procambarus* sp.). Chemosphere.

[26] Oc S., Eraslan S., Kirdar B. (2020). Dynamic transcriptional response of *Saccharomyces cerevisiae* cells to copper. Sci. Rep..

[27] Shi H., Jiang Y., Yang Y., Peng Y., Li C. (2020). Copper metabolism in *Saccharomyces cerevisiae*: An update. Biometals.

[28] Sherman F. (2002). Getting started with yeast. Methods Enzymol..

[29] Labbé S., Zhu Z., Thiele D.J. (1997). Copper-specific transcriptional repression of yeast genes encoding critical components in the copper transport pathway. J. Biol. Chem..

[30] Martins L.J., Jensen L.T., Simon J.R., Keller G.L., Winge D.R. (1998). Metalloregulation of *FRE1* and *FRE2* homologs in *Saccharomyces cerevisiae*. J. Biol. Chem..

[31] Jungmann J., Reins H.A., Lee J., Romeo A., Hassett R., Kosman D., Jentsch S. (1993). MAC1, a nuclear regulatory protein related to Cu-dependent transcription factors is involved in Cu/Fe utilization and stress resistance in yeast. EMBO J..

[32] Dix D., Bridgham J., Broderius M., Eide D. (1997). Characterization of the FET4 protein of yeast. Evidence for a direct role in the transport of iron. J. Biol. Chem..

[33] Liu X.F., Supek F., Nelson N., Culotta V.C. (1997). Negative control of heavy metal uptake by the *Saccharomyces cerevisiae BSD2* gene. J. Biol. Chem..

[34] Jensen L.T., Ajua-Alemanji M., Culotta V.C. (2003). The *Saccharomyces cerevisiae* high affinity phosphate transporter encoded by *PHO84* also functions in manganese homeostasis. J. Biol. Chem..

[35] Ofiteru A.M., Ruta L.L., Rotaru C., Dumitru I., Ene C.D., Neagoe A., Farcasanu I.C. (2012). Overexpression of the *PHO84* gene causes heavy metal accumulation and induces Ire1p-dependent unfolded protein response in *Saccharomyces cerevisiae* cells. Appl. Microbiol. Biotechnol..

[36] Kirchman P.A., Botta G. (2007). Copper supplementation increases yeast life span under conditions requiring respiratory metabolism. Mech. Ageing Dev..

[37] Lapinskas P.J., Cunningham K.W., Liu X.F., Fink G.R., Culotta V.C. (1995). Mutations in *PMR1* suppress oxidative damage in yeast cells lacking superoxide dismutase. Mol. Cell. Biol..

[38] Farcasanu I.C., Hirata D., Tsuchiya E., Mizuta K., Miyakawa T. (1999). Involvement of thioredoxin peroxidase type II (Ahp1p) of *Saccharomyces cerevisiae* in Mn^2+^ homeostasis. Biosci. Biotechnol. Biochem..

[39] Yu W., Farrell R.A., Stillman D.J., Winge D.R. (1996). Identification of *SLF1* as a new copper homeostasis gene involved in copper sulfide mineralization in *Saccharomyces cerevisiae*. Mol. Cell. Biol..

[40] Linderholm A.L., Findleton C.L., Kumar G., Hong Y., Bisson L.F. (2008). Identification of genes affecting hydrogen sulfide formation in *Saccharomyces cerevisiae*. Appl. Environ. Microbiol..

[41] Kim H.S., Huh J., Riles L., Reyes A., Fay J.C. (2012). A noncomplementation screen for quantitative trait alleles in *Saccharomyces cerevisiae*. G3.

[42] Ruta L.L., Kissen R., Nicolau I., Neagoe A.D., Petrescu A.J., Bones A.M., Farcasanu I.C. (2017). Heavy metal accumulation by *Saccharomyces cerevisiae* cells armed with metal binding hexapeptides targeted to the inner face of the plasma membrane. Appl. Microbiol. Biotechnol..

[43] Amberg D.C., Burke D.J., Strathern J.N., Burke D., Dawson D., Stearns T. (2005). Measuring yeast cell density by spectrophotometry. Methods in Yeast Genetics. A Cold Spring Harbor Laboratory Course Manual.

[44] Wofford J.D., Park J., McCormick S.P., Chakrabarti M., Lindahl P.A. (2016). Ferric ions accumulate in the walls of metabolically inactivating *Saccharomyces cerevisiae* cells and are reductively mobilized during reactivation. Metallomics.

[45] Ruta L.L., Popa C.V., Nicolau I., Farcasanu I.C. (2016). Calcium signaling and copper toxicity in *Saccharomyces cerevisiae* cells. Environ. Sci. Pollut. Res. Int..

[46] Marczenko Z., Balcerzak M., Kloczko E. (2000). Copper. Separation, Preconcentration and Spectrophotometry in Inorganic Analysis.

[47] Bradford M.M. (1976). A rapid and sensitive method for the quantitation of microgram quantities of protein utilizing the principle of protein-dye binding. Anal. Biochem..

[48] Kinoshita T., Kiso K., Le D.Q., Shiigi H., Nagaoka T. (2016). Light-scattering characteristics of metal nanoparticles on a single bacterial cell. Anal. Sci..

[49] Weinhandl K., Winkler M., Glieder A., Camattari A. (2014). Carbon source dependent promoters in yeasts. Microb. Cell Fact..

[50] Ruta L.L., Popa C.V., Nicolau I., Farcasanu I.C. (2018). Epigallocatechin-3-*O*-gallate, the main green tea component, is toxic to *Saccharomyces cerevisiae* cells lacking the Fet3/Ftr1. Food Chem..

[51] Xiao Z., Loughlin F., George G.N., Howlett G.J., Wedd A.G. (2004). C-terminal domain of the membrane copper transporter Ctr1 from *Saccharomyces cerevisiae* binds four Cu(I) ions as a cuprous-thiolate polynuclear cluster: Sub-femtomolar Cu(I) affinity of three proteins involved in copper trafficking. J. Am. Chem. Soc..

[52] Palacios O., Atrian S., Capdevila M. (2011). Zn- and Cu-thioneins: A functional classification. J. Biol. Inorg. Chem..

[53] Oliveira R.P., Basso L.C., Junior A.P., Penna T.C., Del Borghi M., Converti A. (2012). Response of *Saccharomyces cerevisiae* to cadmium and nickel stress: The use of the sugar cane vinasse as a potential mitigator. Biol. Trace Elem. Res..

[54] Zhang D., Ma N., Guo Z., Chen P., Ma R., Sun X., Wang D., Wang J., Xu Y. (2019). Improved cadmium resistance and removal capacity in *Pichia kudriavzevii* A16 by sucrose preincubation. J. Basic Microbiol..

[55] Farcasanu I.C., Mizunuma M., Nishiyama F., Miyakawa T. (2005). Role of L-histidine in conferring tolerance to Ni^2+^ in *Sacchromyces cerevisiae* cells. Biosci. Biotechnol. Biochem..

[56] Xu Q., Wu M., Hu J., Gao M.T. (2016). Effects of nitrogen sources and metal ions on ethanol fermentation with cadmium-containing medium. J. Basic Microbiol..

[57] Oprea E., Ruta L.L., Nicolau I., Popa C.V., Neagoe A.D., Farcasanu I.C. (2014). *Vaccinium corymbosum* L. (blueberry) extracts exhibit protective action against cadmium toxicity in *Saccharomyces cerevisiae* cells. Food Chem..

[58] Jia B., Liu X., Zhan J., Li J., Huang W. (2015). The effect of proanthocyanidins on growth and alcoholic fermentation of wine yeast under copper stress. J. Food Sci..

[59] Zimdars S., Schrage L., Sommer S., Schieber A., Weber F. (2019). Influence of glutathione on yeast fermentation efficiency under copper stress. J. Agric. Food Chem..

