# Supplementary material for: Saccharomyces cerevisiae Concentrates Subtoxic Copper onto Cell Wall from Solid Media Containing Reducing Sugars as Carbon Source
Source: Bioengineering (Basel). 2021 Mar 6;8(3):36. doi: 10.3390/bioengineering8030036 (PMC8000517; doi:10.3390/bioengineering8030036)
Supplement: Supplementary file 1 [file bioengineering-08-00036-s001.pdf]

bioengineering

MDPI

Article

Saccharomyces cerevisiae Concentrates Subtoxic Copper onto Cell Wall from Solid Media Containing Reducing Sugars as Carbon Source

Lavinia L. Ruta and Ileana C. Farcasanu *

University of Bucharest, Faculty of Chemistry, Department of Organic Chemistry, Biochemistry and Catalysis, Sos. Panduri 90-92, 050663 Bucharest, Romania; lavinia.ruta@chimie.unibuc.ro
* Correspondence: ileana.farcasanu@chimie.unibuc.ro; Tel.: +40-721-067-169

Abstract: Copper is essential for life, but it can be deleterious in concentrations that surpass the physiological limits. Copper pollution is related to widespread human activities, such as viticulture and wine production. To unravel aspects of how organisms cope with copper insults, we used *Saccharomyces cerevisiae* as a model for adaptation to high but subtoxic concentrations of copper. We found that *S. cerevisiae* cells could tolerate high copper concentration by forming deposits on the cell wall and that the copper-containing deposits accumulated predominantly when cells were grown statically on media prepared with reducing sugars (glucose, galactose) as sole carbon source, but not on media containing nonreducing carbon sources, such as glycerol or lactate. Exposing cells to copper in liquid media under strong agitation prevented the formation of copper-containing deposits at the cell wall. Disruption of low-affinity copper intake through the plasma membrane increased the potential of the cell to form copper deposits on the cell surface. These results imply that biotechnology problems caused by high copper concentration can be tackled by selecting yeast strains and conditions to allow the removal of excess copper from various contaminated sites in the forms of solid deposits which do not penetrate the cell.

Keywords: copper; *Saccharomyces cerevisiae*; carbon source; coloration; extracellular deposit

Citation: Ruta, L.L.; Farcasanu, I.C. *Saccharomyces cerevisiae* Concentrates Subtoxic Copper onto Cell Wall from Solid Media Containing Reducing Sugars as Carbon Source. *Bioengineering* **2021**, *8*, 36. https://doi.org/10.3390/bioengineering8030036

Academic Editor: Eva Garcia Ruiz

Received: 12 December 2020
Accepted: 3 March 2021
Published: 6 March 2021

Publisher's Note: MDPI stays neutral with regard to jurisdictional claims in published maps and institutional affiliations.

Copyright: © 2021 by the authors. Licensee MDPI, Basel, Switzerland. This article is an open access article distributed under the terms and conditions of the Creative Commons Attribution (CC BY) license (http://creativecommons.org/licenses/by/4.0/).

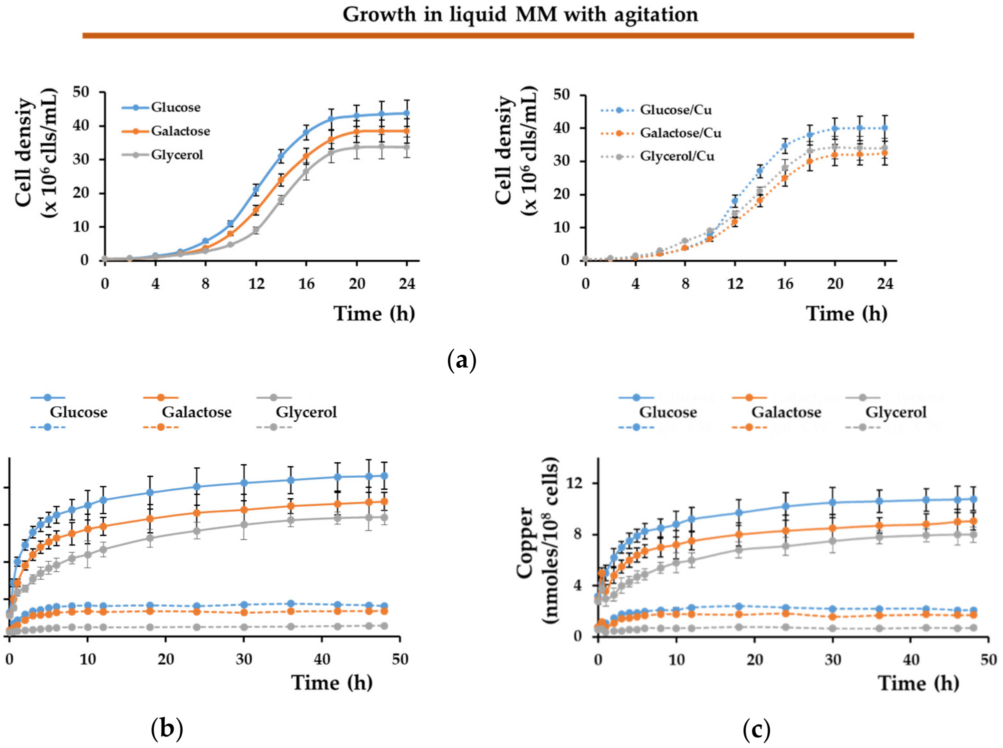

Figure S1. Copper accumulation by yeast cell grown with agitation in MM liquid media. Cells of wild type strain BY4741 were inoculated (5×10^5 cells/mL) in MM/Glc (Glucose), MM/Gal (GalacTable 28. °C, 200 rpm). (**a**) Cell proliferation was determined spectrophotometrically (OD_{660}) in the absence (left pannel) or presence (right pannel) of 0.1 mM $CuCl_2$. (**b**,**c**) Copper associated with whole cells (full lines) or with cell walls (dotted lines) was determined as described in *Materials and methods* and normalized to total cell protein (**b**) or to 10^8 cells (**c**). Values are mean ± SEM of triplicate determinations done on three biological repeats.

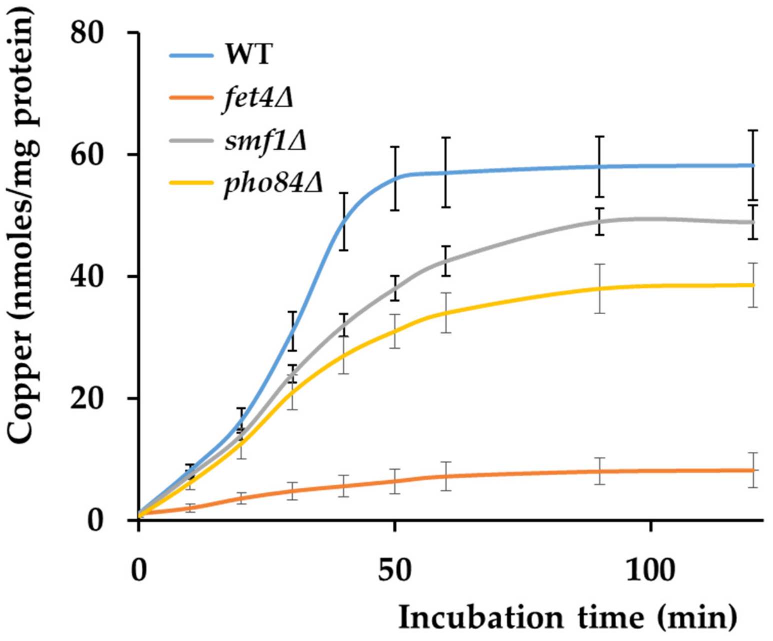

Figure S2. Copper accumulation by yeast mutants grown with agitation (28 °C, 200 rpm) in MM/Glc liquid media. Cell suspensions of strain BY4741 (WT) and of isogenic strains *fet4Δ*, *smf1Δ*, and *pho84Δ* were incubated with 0.5 mM $CuCl_2$. Cells were harvested by centrifugation at various times and processed for copper assay, as described in *Materials and methods*. Values are mean ± SEM of triplicate determination done on three biological repeats.

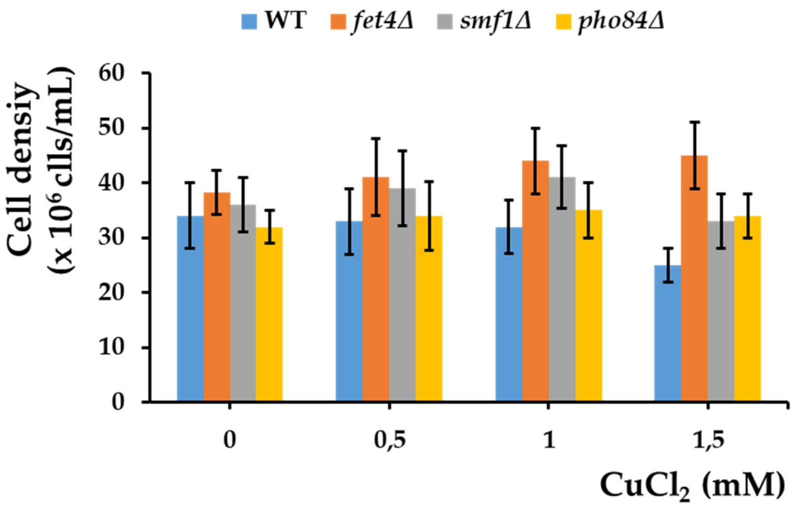

Figure S3. Effect of copper on the growth of yeast mutants with defects in metal transport across plasma membrane. Cell suspensions of strain BY4741 (WT) and of isogenic strains *fet4Δ*, *smf1Δ*, and *pho84Δ* were inoculated (5×10^5 cells/mL) in MM/Glc and incubated for 2-4 h (28 °C, 200 rpm) before $CuCl_2$ was added to various concentrations. Cell proliferation was determined spectrophotometrically (OD_{660}) 24 h following copper addition. Values are mean ± SEM of triplicate determination done on three biological repeats.
